# MPPT control of a solar pumping system based five-phase impedance source inverter fed induction motor

**DOI:** 10.1371/journal.pone.0295365

**Published:** 2024-01-18

**Authors:** Ahmed M. Hassan, Abd El-Wahab Hassan, Z. M. S. Elbarbary, Saad F. Al-Gahtani, Ahmed I. Omar, Mohamed Eladly Metwally

**Affiliations:** 1 Faculty of Engineering, Electrical Engineering Department, Benha University, Banha, Cairo, Egypt; 2 Department of Electrical Power and Machines Engineering, Higher Institute of Engineering, El-Shorouk Academy, El-Shorouk City, Cairo, Egypt; 3 Faculty of Engineering, Department of Electrical Power and Machines Engineering, Tanta University, Tanta, Egypt; 4 Electrical Engineering Department, College of Engineering, King Khalid University, Abha, Saudi Arabia; Vardhaman College of Engineering, INDIA

## Abstract

This paper presents a control method for a system composed of a photovoltaic (PV) array, five-phase impedance source inverter, five-phase induction motor and centrifugal pump. This method is based on controlling the motor speed to control the pump power as the insolation level or temperature change to attain the maximum power extraction from the PV-array. The motor speed is controlled by using artificial neural network (ANN) which is trained to provide the desired inverter frequency and modulation index at any insolation level and temperature to attain the maximum PV operating power. The data of the neural network are based on the operation of the induction motor at constant air gap flux and perturb and observe method for maximum power point tracking. Simulation results are obtained using MATLAB Simulink to verify the proposed control method.

## Introduction

The use of a photovoltaic (PV) array as a source of electrical energy has many benefits. It is both noiseless and dependable, while also being environmentally friendly and simple to install. Although it requires little maintenance, its initial installation costs can be high, and its power conversion efficiency can be low. Nevertheless, photovoltaic pumping is an encouraging application of photovoltaic energy. By storing solar energy as potential energy in a water reservoir, energy can be consumed on demand. This eliminates the need for large, expensive, and heavy banks of lead acid batteries, which have a significantly shorter lifespan than photovoltaic panels. However, it is crucial to maintain an efficient power conversion chain from panels to mechanical pump, despite the lack of batteries [1]. Solar-powered pumping systems have become increasingly popular due to their numerous benefits over traditional diesel pumps. These advantages include lower operational and maintenance costs, as well as improved reliability. Since the demand for water is highest during the day, solar pumping systems are well-suited to meet this need. However, they are also subject to weather and environmental conditions that can affect their performance. This means that intermittent power shortages may occur, particularly on cloudy days. To reduce costs and improve efficiency, it is important to operate the PV source at its maximum power point. This will prevent the need for oversized PV panels and improve the reliability of the entire system [2].

There have been several studies that have dealt with the solar pumping systems [1–14]. A field-oriented control method for a battery less solar pumping system composed of a push-pull converter and three-phase induction motor was presented in [1]. In [2], a field-oriented control method for a standalone solar powered water pumping system employing permanent magnet synchronous motor was presented. The effect of voltage and load variation on a solar pumping system employing three-phase induction motor was investigated via experimental results in [4]. A cost effective solar pumping system employing brushless direct current motor was presented in [5]. Control methods for a solar pumping system employing an open-end winding induction motor and dual inverter were presented in [6,7]. In [8], a control method was introduced for a single stage solar pumping system utilizing brushless direct current motor and a common voltage source inverter. Design and control of a solar pumping system employing switched reluctance motor and dual supply buck-boost converter was presented in [9]. In [10], a CUK-SEPIC converter for solar water pumping system was presented. This system was operated at maximum power point using a hybrid gravitational search algorithm and particle swarm optimization. The motor used in this system was switched reluctance motor. In [11], a hybrid photovoltaic-wind water pumping system was presented. This system employed a CUK converter, battery bank, permanent magnet synchronous generator, rectifier, three-phase inverter, and three-phase induction motor. The system was controlled using a modified artificial bee colony based on maximum power point tracking algorithm. In [12], a modified hybrid grey wolf optimization fuzzy logic controller based maximum power point tracking for a solar pumping system employing switched reluctance motor was presented. An optimized hybrid firefly based ant colony optimization as maximum power point tracking for a solar pumping system utilizing permanent magnet synchronous motor was presented in [13]. In [14], a hybrid firefly algorithm-particle swarm optimization based maximum power point tracking for a solar pumping system employing three-phase induction motor was presented. However, there is not any research that has dealt with solar pumping systems based on five-phase impedance source inverters and five-phase induction motors. The impedance source inverter has many advantages, such as superior efficiency and compact structure. The impedance source inverter is preferred over conventional two-stage inverters because of its fewer power devices [15,16].

Electric machines utilizing multiphase inverter technology are currently preferred over conventional three-phase machines and inverters. This is due to several advantages such as the reduction of torque pulsation amplitude and increase of frequency of these pulsations, higher torque density, decreased per phase rotor harmonic current distortion without the need for an increase in voltage per phase, lower dc-link current ripples, better noise characteristics, and higher reliability with increased fault tolerance [17–21].

In this paper, a control method is proposed for a water pumping system composed of PV array, five-phase impedance source inverter (ZSI), five-phase induction motor and centrifugal pump. This method is based on controlling the motor speed to control the pump power as the insolation level or temperature change to attain the maximum power point. The motor speed is controlled by using an artificial neural network which is trained to provide the desired inverter frequency and modulation index at any insolation level and temperature to attain the maximum PV operating power. The data of the neural network are based on the operation of the induction motor at constant air gap flux and P&O method for MPPT. The main contribution of this work can be summed up as follows:

- A new standalone solar pumping system is proposed. This system is composed of PV array, five-phase ZSI, five-phase induction motor and centrifugal pump.
- A new control method for the standalone solar pumping system is proposed. This control method achieved maximum utilization of solar energy.
- The artificial neural network is employed in the control methodology to improve the system performance.
- The system is simulated using MATLAB SIMULINK to prove the validity of the proposed control methodology for the proposed solar pumping system.

## System modelling

In this section, each part of the system is modelled in the following subsections.

### PV array model

**Fig 1:**
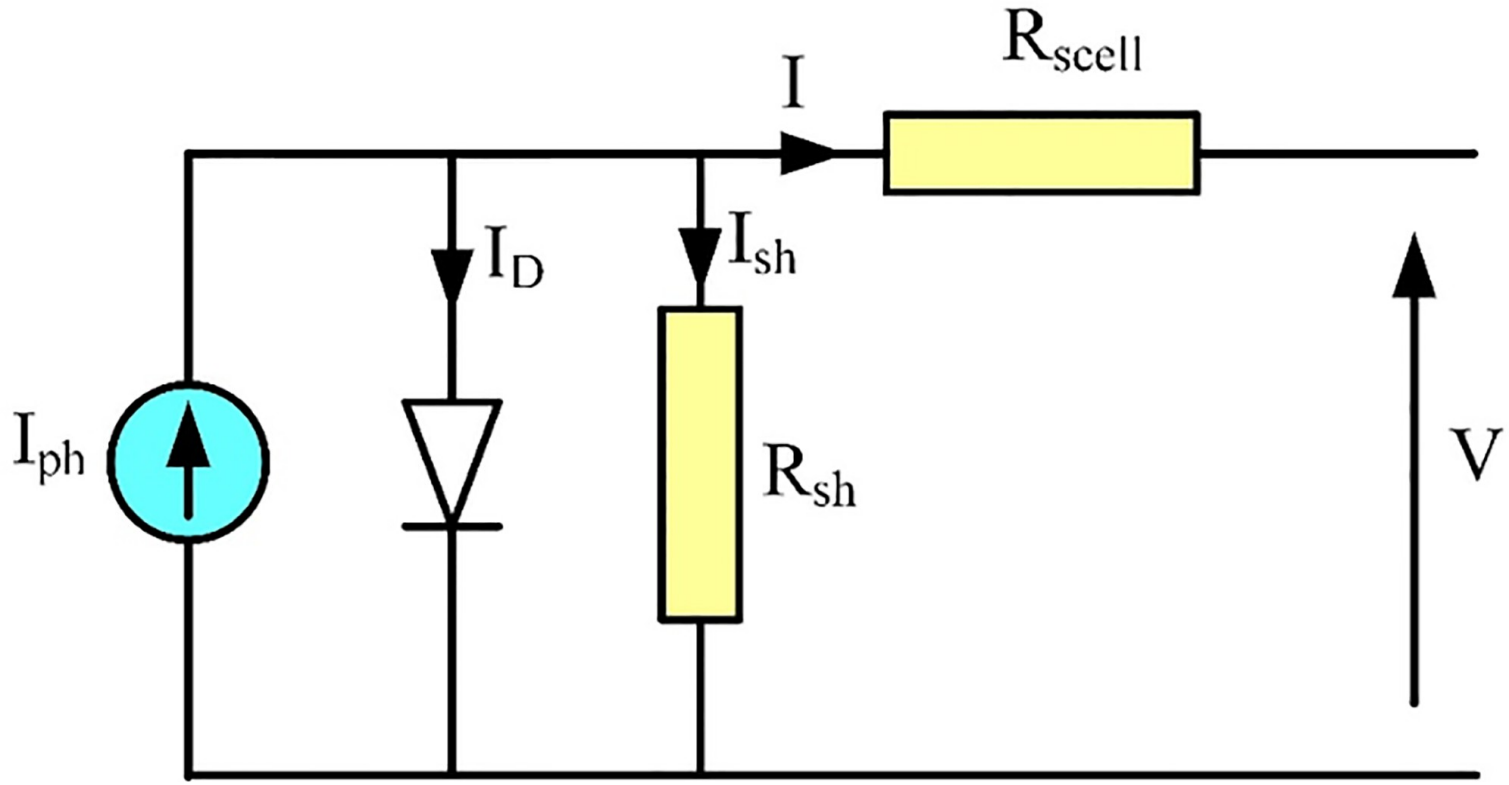
Solar cell equivalent circuit.

The PV array is composed of several modules and each module consists of a number of solar cells [22]. The electrical equivalent circuit of a solar cell is shown in Fig 1 [23,24].

The expression relating the PV cell output voltage, V, and output current, I, is given by [23]:

$$I=I_{ph}-I_{o}(e^{\Lambda .(V+I.R_{scell})}-1)-\frac{V+I.R_{scell}}{R_{sh}}$$ (1)

Where $\Lambda =\frac{q}{AkT_{cell}}$, *I*_*ph*_ is the cell photo current, *I*_*o*_ is the cell reverse saturation current, *q* is the electron charge, *A* is an ideality factor, k is Boltzmann’s constant and *T*_*cell*_ is the absolute cell temperature [25]. When the PV array is formed from several parallel strings, *N*_*p*_, and each string consists of number of series connected modules, *N*_*m*_, and each module consists of number of series connected cells, *N*_*s*_, the expression relating the PV array output voltage, *V*_*g*_, to its output current, *I*_*g*_, can be obtained from:

$$I_{g}=I_{phg}-I_{og}(e^{\Lambda_{g}.(V_{g}+I_{g}.R_{sg})}-1)-\frac{V_{g}+I_{g}.R_{sg}}{R_{shg}}$$ (2)

where *N*_*sg*_ = *N*_*m*_.*N*_*s*_, *I*_*phg*_ = *I*_*ph*_.*N*_*p*_, *I*_*og*_ = *I*_*o*_.*N*_*p*_, $R_{sg}=R_{scell}.\frac{N_{sg}}{N_{p}}$, $R_{shg}=R_{sh}.\frac{N_{sg}}{N_{p}}$, $\Lambda_{g}=\frac{\Lambda }{N_{sg}}$, *I*_*phg*_ is the PV array photocurrent, *I*_*og*_ is the PV array reverse saturation current, *R*_*sg*_ is the PV array series resistance, *R*_*shg*_ is the PV array shunt resistance and Λ_*g*_ is the factor of the PV array [26,27].

In order to use Eq (2) at any insolation level, the per unit insolation level, *G*, is included into the equation as follows:

$$I_{g}=G.I_{phg}-I_{og}(e^{\Lambda_{g}(V_{g}+I_{g}.R_{sg})}-1)-\frac{V_{g}+I_{g}.R_{sg}}{R_{shg}}$$ (3)

where in Eq (4), *I*_*phg*_ is the array photocurrent at an insolation level of 100% (1000 W/m^2^).

### Five-phase impedance source inverter model

**Fig 2:**
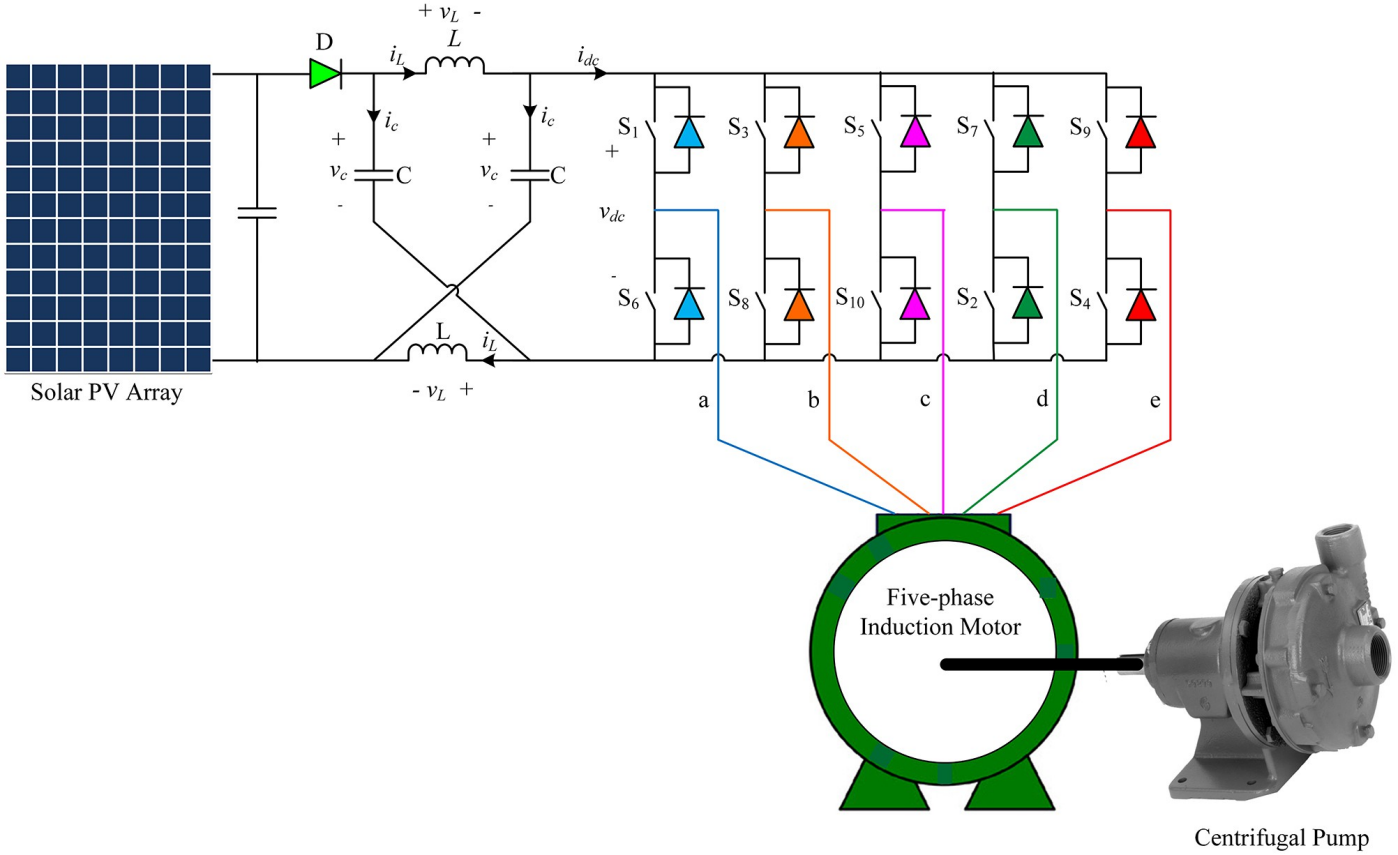
Solar pumping system under consideration.

The five-phase impedance source inverter includes impedance network that connects the main converter circuit with a DC source, PV array [28]. This network consists of two inductors, with equal inductance (L), and two capacitors, with equal capacitance (C), Fig 2. The diode in Fig 2 prevents the reverse current flow. The most important feature of this inverter is that its output voltage can be controlled from zero to infinity, i.e., the impedance source inverter is a buck-boost inverter. Either the conventional voltage source inverter or current source inverter cannot attain this feature [29].

The five-phase impedance source inverter shown in Fig 2 has three groups of switching states. The first group is called active states where the load is connected directly to the supply. The second group is called null states where either all the upper or all the lower switches are conducted simultaneously. The third group is called shoot-through state in which the dc-link terminals are shorted by gating both the upper and lower switches of at least one inverter leg. During the shoot through state the capacitor voltage is boosted by receiving the energy from the inductor. The total number of shoot through states is 31 states [30].

The impedance-source network voltage of the impedance source five-phase should verify equal inductors voltage, *v*_*L*_, and equal capacitors voltages, *v*_*c*_ [29]. In a sampling period *T*, the inverter bridge enters the shoot-through state for a duration *T*_*o*_. During this time, the diode (*D*) in the power circuit (Fig 2) becomes reverse-biased, and the PV source is isolated from the Z-network. The inductors are charged by the Z-source capacitors in this mode, which is included in the conventional zero periods (*T*_*Z*_) of every switching cycle (*T*). The shoot-through interval or its duty cycle is determined by the necessary voltage boost. However, it’s worth noting that the shoot-through interval is only a small fraction of the switching cycle, requiring a relatively small capacitor to suppress the voltage [29]. The voltage equation of the Z-network can be expressed as follows [29].


$$v_{L}=V_{PV}-v_{C}\;$$ (4)

When the output voltage of the PV array, denoted as *V*_*PV*_, matches the DC link voltage (*v*_*dc*_), the two voltages are equal.


$$v_{dc}=v_{C}-v_{L}=2v_{C}-V_{PV}$$ (5)

Eq (5) provides a way to represent the maximum DC link voltage present on the inverter bridge.

$$v_{dc}=v_{C}-v_{L}=2v_{C}-V_{PV}=\frac{1}{1-2D_{0}}V_{PV}=BV_{PV}$$ (6)

Where *B* represents the boost factor, while *D*_0_ is the shoot-through duty ratio and is defined as the ratio of the time during which both switching devices in a converter are on simultaneously to the total switching period and is equal to $\left(\frac{T_{o}}{T}\right)$.

The relationship between the boost factor (B) and the shoot-through duty ratio is given by the following equation:

$$B=\frac{1}{1-2\left(\frac{T_{o}}{T}\right)}=\frac{1}{1-2D_{0}}$$ (7)

In the steady state of non-boost mode operation, the voltage across the Z-source capacitor equals the voltage across the DC-link of the ZSI. To put it differently,

$$V_{c1}=V_{c2}=V_{c}=\frac{1-\left(\frac{T_{o}}{T}\right)}{1-2\left(\frac{T_{o}}{T}\right)}V_{PV}=\frac{1-D_{0}}{1-2D_{0}}V_{PV}=\frac{1+B}{2}V_{PV}=V_{PV}{}^{*}$$ (8)

The maximum power point voltage *V*_PV_*of the PV panel determines the AC output voltage control in the inverter. The modulation index variation enables control over the peak value of the AC output voltage, which can be mathematically represented.

$$v_{ac}=m.B*\frac{V_{PV}}{2}$$ (9)

where *m* is the modulation index.

There are several types of modulation techniques used to control the inverter. The most conventional modulation techniques are simple boost control, maximum boost control, maximum boost control with third harmonic injection, and modified space vector pulse width modulation control [31]. The simple boost control modulation technique is adopted in this study.

### Simple boost control modulation technique

**Fig 3:**
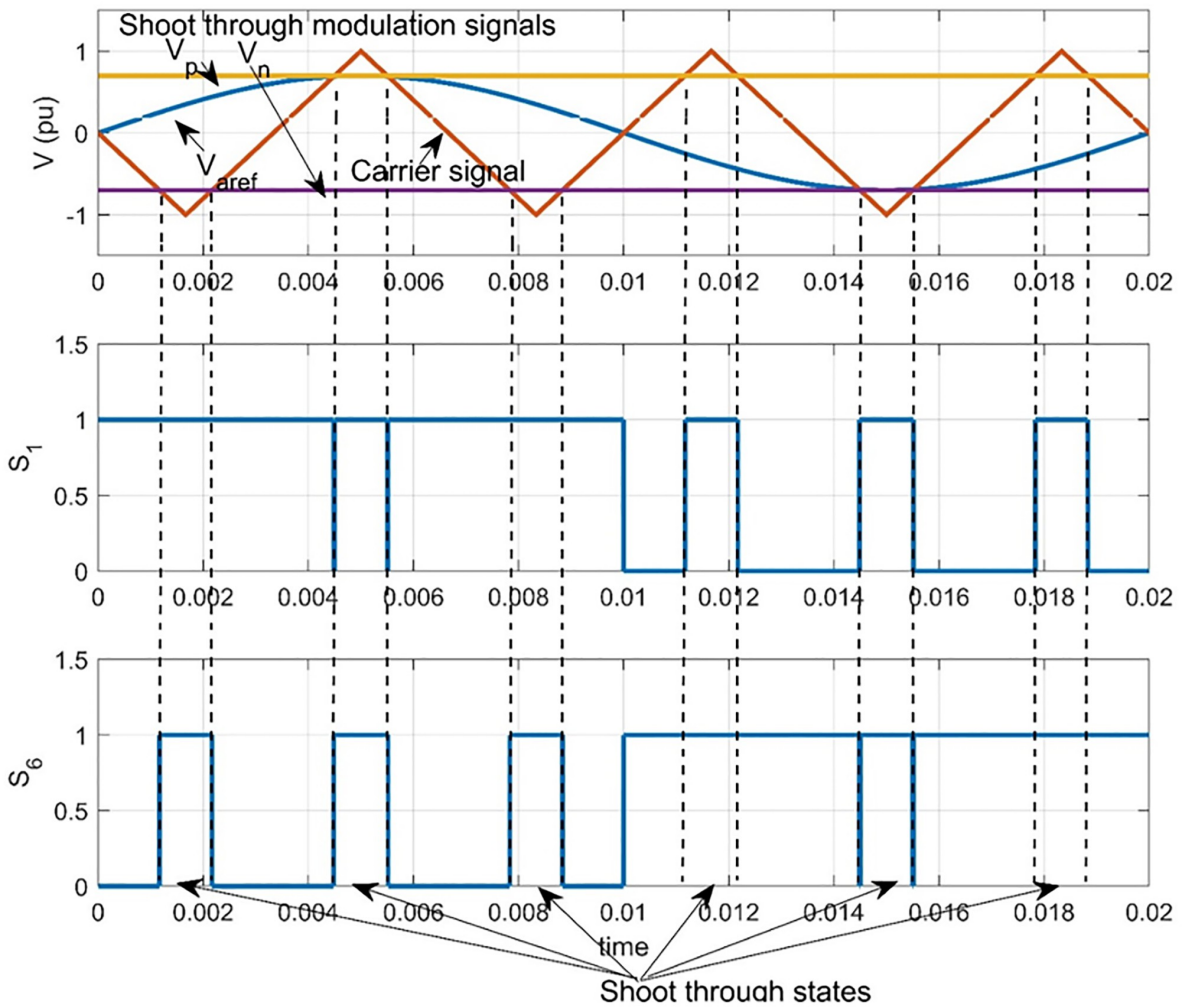
Simple boost modulation technique.

This technique employs two values Vp and Vn as shoot-through modulating signals. These signals have positive and negative peak values of five-phase sinusoidal reference signals as shown in Fig 3 [30]. For a unity carrier signal height, Vp corresponds to value of m [32]. Shoot through occurs only when carrier signal is greater than Vp or lower than Vn, otherwise it operates just as traditional sinusoidal pulse width modulation (SPWM). From Fig 3, shoot-through duty ratio Do is related to modulation index m as follows:

$$D_{o}=1-\text{m}$$ (10)

Substituting Eq (10) into Eq (7), we can obtain the boost factor B as follows:

$$B=\frac{1}{2\text{m}-1}$$ (11)

Substituting Eq (11) into Eq (9), the peak value can be obtained from:

$$v_{ac}=\frac{\text{m}}{2\text{m}-1}*\frac{V_{PV}}{2}$$ (12)

### Five-phase induction motor model

The five-phase induction motor is modeled in terms of the DQ model in the stationary reference frame. The Voltage ABC to DQ Transformation can be obtained from [33]:

$$\left[\begin{array}{c} v_{ds}\\ v_{qs}\\ {v^{\prime }}_{ds}\\ {v^{\prime }}_{qs}\\ v_{0} \end{array}\right]=\sqrt{\frac{2}{5}}\left[\begin{array}{ccccc} 1 & \text{cos}\;\alpha & \text{cos}\;2\alpha & \text{cos}\;3\alpha & \text{cos}\;4\alpha \\ 0 & \text{sin}\;\alpha & \text{sin}\;2\alpha & \text{sin}\;3\alpha & \text{sin}\;4\alpha \\ 1 & \text{cos}\;2\alpha & \text{cos}\;4\alpha & \text{cos}\;6\alpha & \text{cos}\;8\alpha \\ 0 & \text{sin}\;2\alpha & \text{sin}\;4\alpha & \text{sin}\;6\alpha & \text{sin}\;8\alpha \\ \frac{1}{2} & \frac{1}{2} & \frac{1}{2} & \frac{1}{2} & \frac{1}{2} \end{array}\right].\left[\begin{array}{c} v_{as}\\ v_{bs}\\ v_{cs}\\ v_{ds}\\ v_{es} \end{array}\right]$$ (13)

Where α = 2π/5.

The current DQ to ABC Transformation can be obtained from [33]:

$$\left[\begin{array}{c} i_{as}\\ i_{bs}\\ i_{cs}\\ i_{ds}\\ i_{es} \end{array}\right]=\sqrt{\frac{2}{5}}\left[\begin{array}{ccccc} 1 & 0 & 1 & 0 & 1\\ \text{cos}\;\alpha & \text{sin}\;\alpha & \text{cos}\;2\alpha & \text{sin}\;2\alpha & 1\\ \text{cos}\;2\alpha & \text{sin}\;2\alpha & \text{cos}\;4\alpha & \text{sin}\;4\alpha & 1\\ \text{cos}\;3\alpha & \text{sin}\;3\alpha & \text{cos}\;6\alpha & \text{sin}\;6\alpha & 1\\ \text{cos}\;4\alpha & \text{sin}\;4\alpha & \text{cos}\;8\alpha & \text{sin}\;8\alpha & 1 \end{array}\right].\left[\begin{array}{c} i_{ds}\\ i_{qs}\\ {i^{\prime }}_{ds}\\ {i^{\prime }}_{qs}\\ i_{0s} \end{array}\right]$$ (14)

The following equation represents the DQ motor voltage equations [33]:

$$\left[\begin{array}{c} v_{ds}\\ v_{qs}\\ 0\\ 0 \end{array}\right]=\left[\begin{array}{cccc} R_{s}+L_{s}D & 0 & M.D & 0\\ 0 & R_{s}+L_{s}D & 0 & M.D\\ M.D & \omega_{me}M & R_{r}+L_{r}D & \omega_{me}L_{r}\\ -\omega_{me}M & M.D & -\omega_{me}L_{r} & R_{r}+L_{r}D \end{array}\right].\left[\begin{array}{c} i_{ds}\\ i_{qs}\\ i_{dr}\\ i_{qr} \end{array}\right]$$ (15)

and

$$\left[\begin{array}{c} v_{ds}^{\prime }\\ v_{qs}^{\prime }\\ 0\\ 0 \end{array}\right]=\left[\begin{array}{cccc} R_{s}+L_{ls}D & 0 & 0 & 0\\ 0 & R_{s}+L_{ls}D & 0 & 0\\ 0 & 0 & R_{r}+L_{lr}D & 0\\ 0 & 0 & 0 & R_{r}+L_{lr}D \end{array}\right].\left[\begin{array}{c} i_{ds}^{\prime }\\ i_{ds}^{\prime }\\ i_{dr}^{\prime }\\ i_{qr}^{\prime } \end{array}\right]$$ (16)

The above motor differential equation can be rewritten in the following form in which the matrix inversion is avoided:

$$D[i]=k\{[A_{1}][v]-[A_{2}].[i]-\omega_{me}[A_{3}].[i]\}$$ (17)

where

$$k=\frac{1}{M^{2}-L_{r}L_{S}},$$

$$\left[A_{1}\right]=\left[\begin{array}{cccccccc} -L_{r} & 0 & M & 0 & 0 & 0 & 0 & 0\\ 0 & -L_{r} & 0 & M & 0 & 0 & 0 & 0\\ M & 0 & -L_{s} & 0 & 0 & 0 & 0 & 0\\ 0 & M & 0 & -L_{s} & 0 & 0 & 0 & 0\\ 0 & 0 & 0 & 0 & \frac{1}{kL_{ls}} & 0 & 0 & 0\\ 0 & 0 & 0 & 0 & 0 & \frac{1}{kL_{ls}} & 0 & 0\\ 0 & 0 & 0 & 0 & 0 & 0 & \frac{1}{kL_{lr}} & 0\\ 0 & 0 & 0 & 0 & 0 & 0 & 0 & \frac{1}{kL_{lr}} \end{array}\right]$$

$$\left[A_{2}\right]=\left[\begin{array}{cccccccc} -L_{r}R_{s} & 0 & MR_{r} & 0 & 0 & 0 & 0 & 0\\ 0 & -L_{r}R_{s} & 0 & MR_{r} & 0 & 0 & 0 & 0\\ MR_{s} & 0 & -L_{s}R_{r} & 0 & 0 & 0 & 0 & 0\\ 0 & MR_{s} & 0 & -L_{s}R_{r} & 0 & 0 & 0 & 0\\ 0 & 0 & 0 & 0 & \frac{R_{s}}{kL_{ls}} & 0 & 0 & 0\\ 0 & 0 & 0 & 0 & 0 & \frac{R_{s}}{kL_{ls}} & 0 & 0\\ 0 & 0 & 0 & 0 & 0 & 0 & \frac{R_{r}}{kL_{lr}} & 0\\ 0 & 0 & 0 & 0 & 0 & 0 & 0 & \frac{R_{r}}{kL_{lr}} \end{array}\right],$$

$$\left[A_{3}\right]=\left[\begin{array}{cccccccc} 0 & M^{2} & 0 & L_{r}M & 0 & 0 & 0 & 0\\ -M^{2} & 0 & -L_{r}M & 0 & 0 & 0 & 0 & 0\\ 0 & -L_{s}M & 0 & -L_{r}L_{s} & 0 & 0 & 0 & 0\\ L_{s}M & 0 & L_{r}L_{s} & 0 & 0 & 0 & 0 & 0\\ 0 & 0 & 0 & 0 & 0 & 0 & 0 & 0\\ 0 & 0 & 0 & 0 & 0 & 0 & 0 & 0\\ 0 & 0 & 0 & 0 & 0 & 0 & 0 & 0\\ 0 & 0 & 0 & 0 & 0 & 0 & 0 & 0 \end{array}\right]$$

The developed torque can be obtained as follows:

$$T_{e}=p.M.[i_{dr}.i_{qs}-i_{qr}.i_{ds}]$$ (18)

When the variation of the motor speed is taken into consideration during the transient period, Eq (13) becomes nonlinear and numerical solution to obtain the motor currents is required. For this purpose, the motor mechanical equation is used. The induction motor mechanical equation is given by:

$$D\omega_{m}=\frac{T_{e}-T_{l}(\omega_{m})}{J}$$ (19)

where *ω*_*m*_ is the motor speed in rad/sec, J is the inertia and *T*_*l*_(*ω*_*m*_) is given by:

$$T_{l}(\omega_{m})=T_{L}+T_{fw}$$ (20)

Where *T*_*L*_ is the load torque, *T*_*fw*_ is the friction and windage torque and *T*_*e*_ is obtained from Eq (18).

### Centrifugal pump model

The centrifugal pump is modeled using its torque-speed equation which is given by the following equation [34]:

$$T_{p}=K_{p}\omega_{m}^{2}$$ (21)

Eq (22) can be used to represent the pump power-speed equation as follows:

$$P_{p}=K_{p}\omega_{m}^{3}$$ (22)

## Control methodology

**Fig 4:**
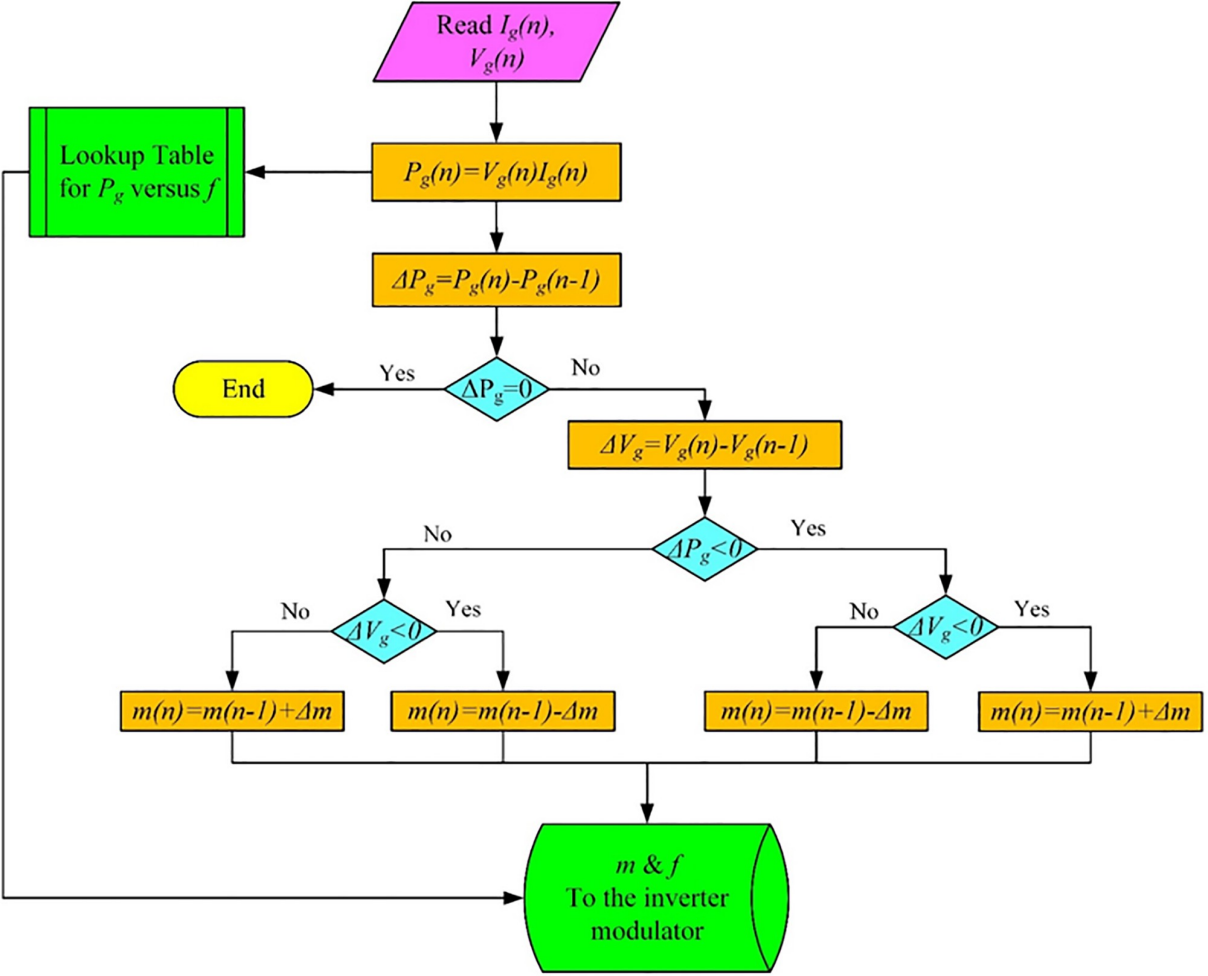
Proposed algorithm to obtain the modulation index and reference frequency.

The main purpose of controlling the system under consideration is to extract the maximum power from the PV array as the insolation level and temperature change [35]. This is achieved by controlling the motor speed as the insolation level changes or temperature [36]. When either the insolation level or temperature changes, the motor frequency and voltage, constant air-gap flux, will be controlled to change motor speed and thus, the pump power, Eq (20), extract the PV array maximum power. The motor frequency is controlled by controlling the inverter reference frequency [37]. The motor voltage is controlled by controlling the modulation index to maintain constant air-gap flux. The PV array maximum power is tracked by perturb and observe method. This method is achieved by changing the modulation index and the inverter frequency until the maximum power is extracted from the PV array. The inverter frequency is obtained from a lookup table whose data are obtained from steady-state analysis for the induction motor which is controlled by operating the motor at constant air-gap flux. The result of the analysis gives data for the motor input power, which is assumed to be equal to PV-array output power, and the corresponding data for the inverter frequency. The algorithm is shown in Fig 4.

The Artificial Neural Network (ANN) is a widely utilized technology in various fields of research and application [38]. It proves beneficial in solving complex problems that cannot be effectively addressed through traditional mathematical approaches [39]. Numerous studies have proposed the application of ANN technique in selecting voltage inverter switches for IM power supply [40,41]. The concept involves replacing the conventional switching table, which determines the inverter states, with a neural selector capable of managing control signals. This architecture incorporates a multilayer neural network that replaces hysteresis comparators and the selection table.

**Fig 5:**
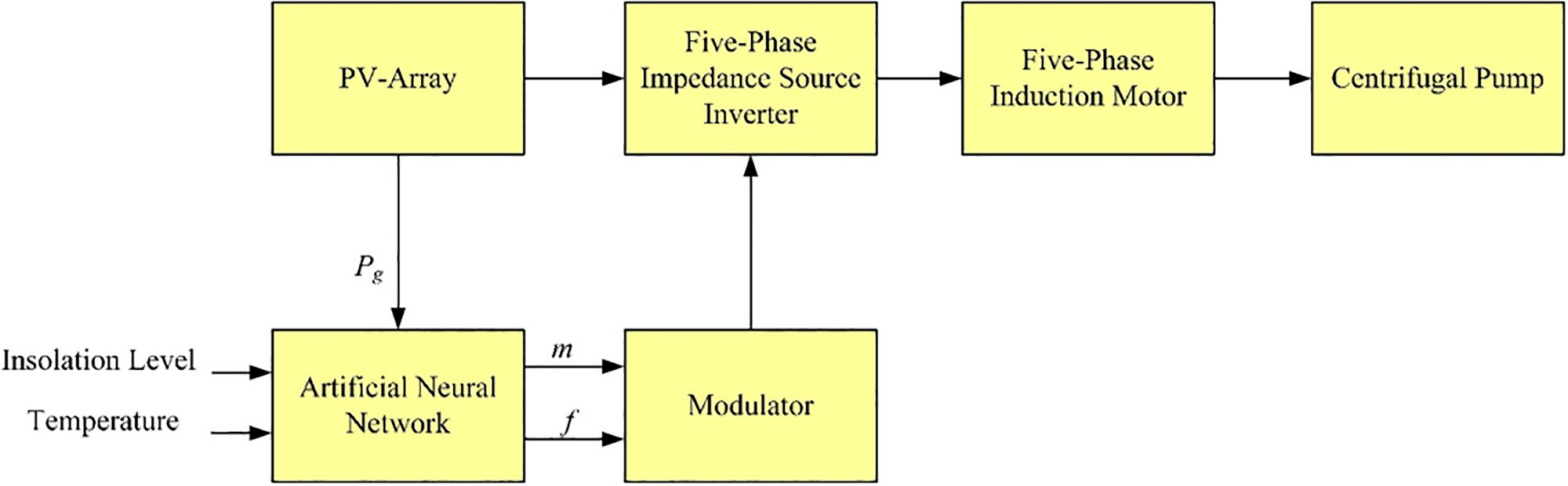
Proposed algorithm using artificial neural network (ANN).

The data obtained from the above-mentioned method are used in an artificial neural network to generate the reference modulation index and reference frequency at a given insolation level and temperature as shown in Fig 5. Training data are generated from Simulink of PV system model obtained from Simulink MATLAB^®^ simulation model designed. In this work the neural network used to emulate the look up table based the desired insolation level and reference frequency.

The structure of the proposed neural network has 3 input nodes, 8 neurons in the hidden layer, and 4 neurons in the output layer. The type of activation function used is a sigmoid function, where the sigmoid functions commonly used in back-propagation networks, in part because it is differentiable. The input data to the proposed neural network are insolation level, temperature, actual generated power. The outputs of the neural network are reference modulation index and reference frequency.

## Simulation results

**Fig 6:**
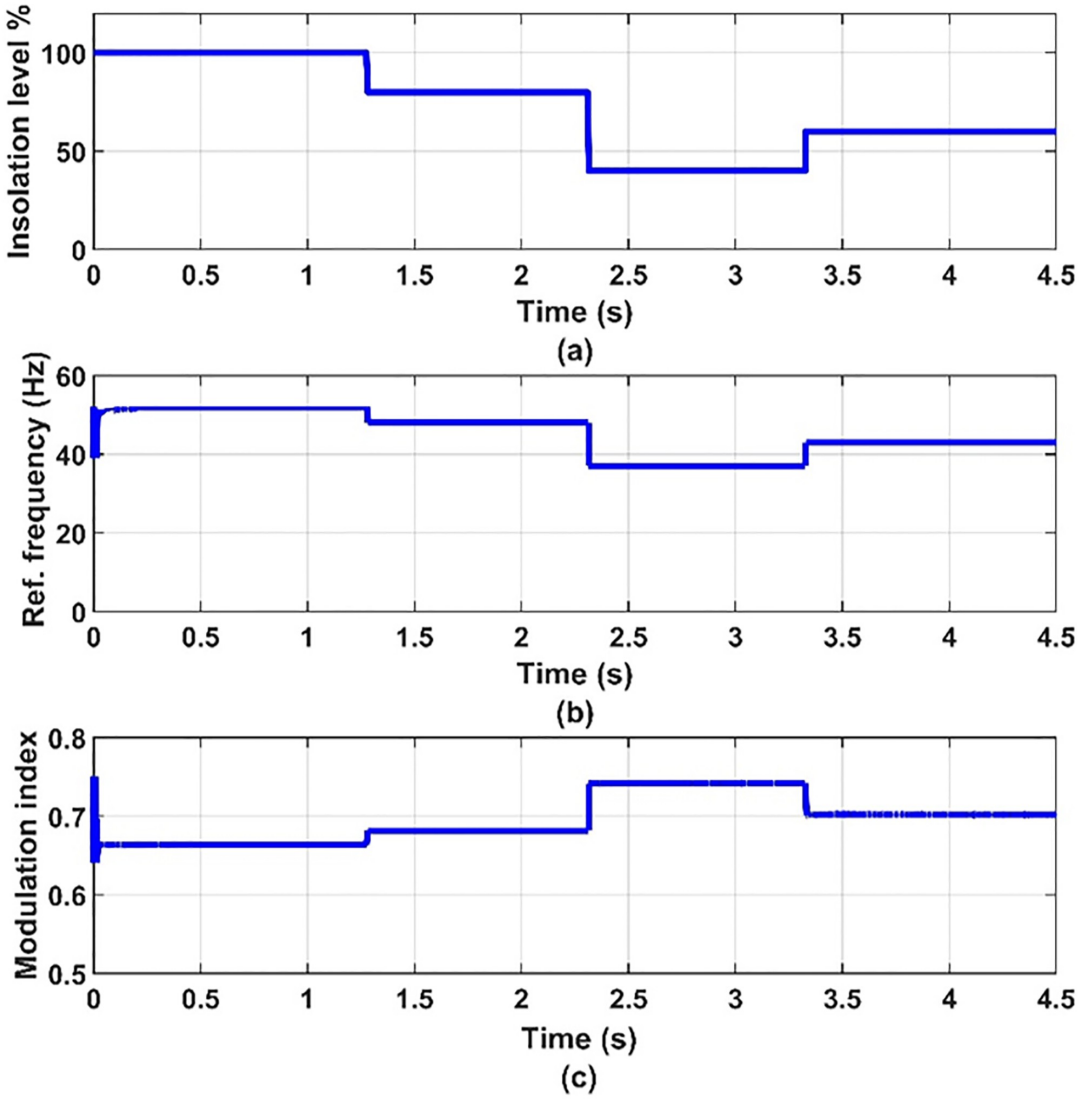
(a) insolation level, (b) inverter reference frequency and (c) modulation index.

**Fig 7:**
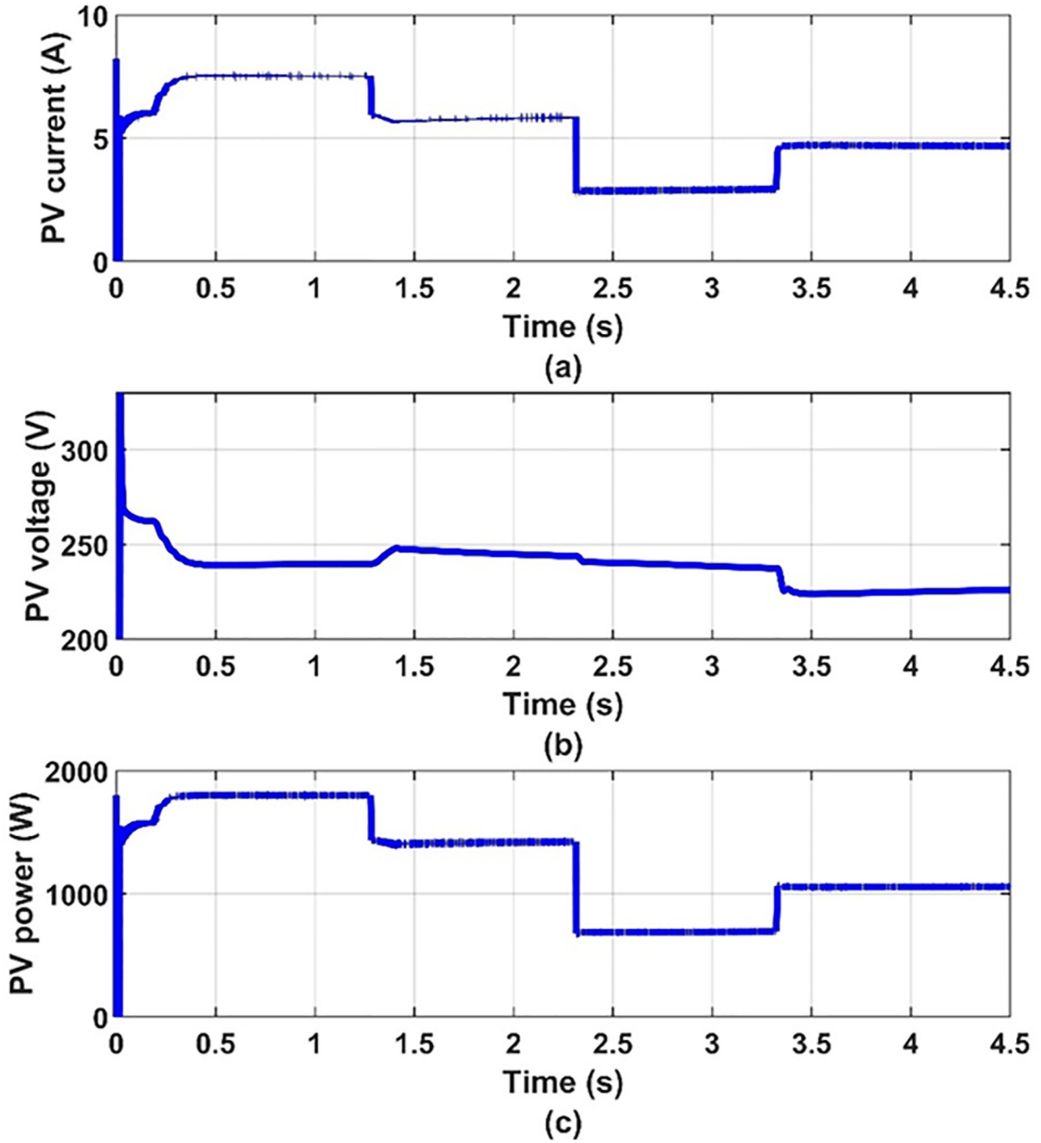
(a) PV-array current, (b) PV-array voltage and (c) PV-array power.

**Fig 8:**
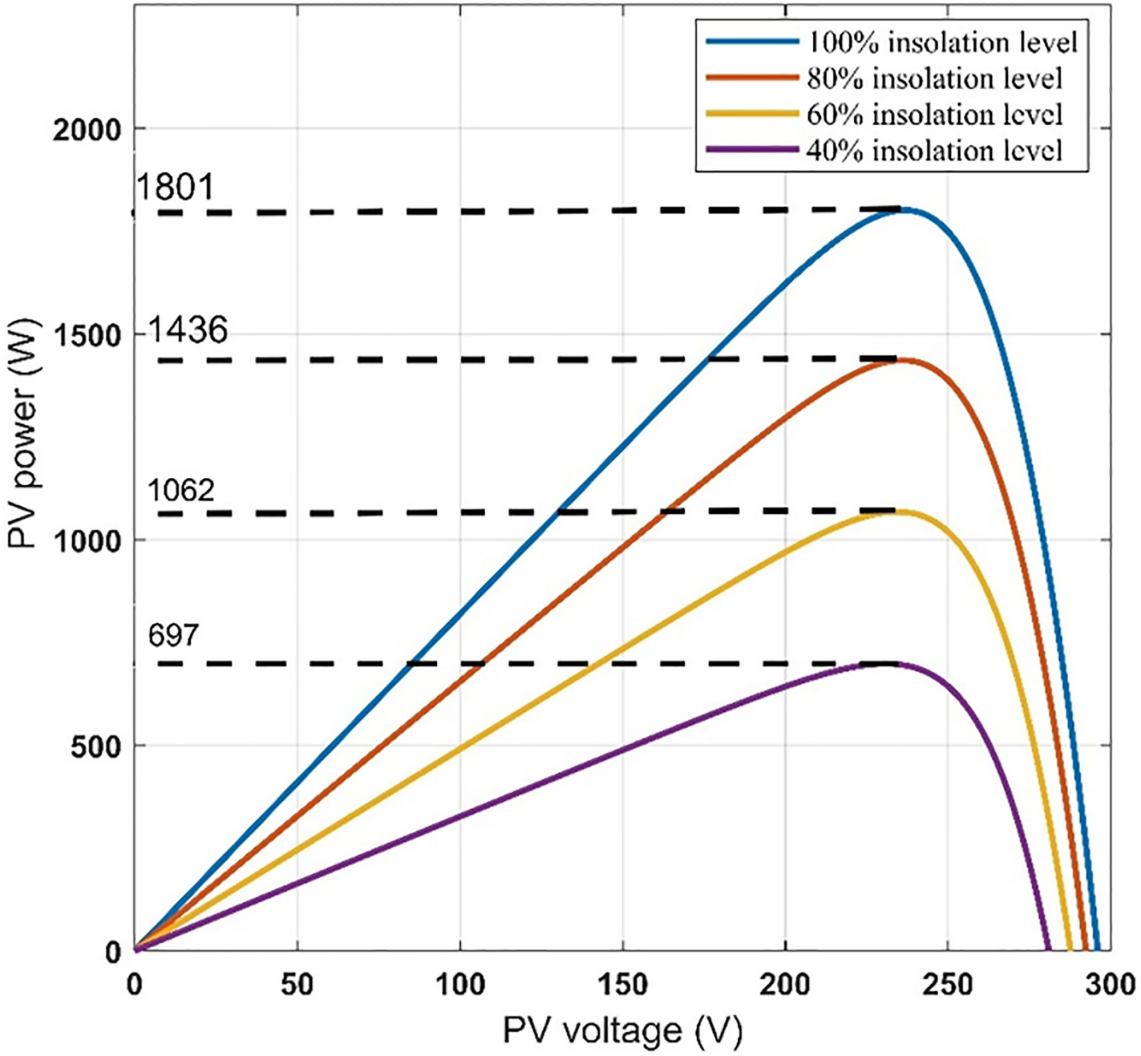
PV-array power-voltage characteristics at different insolation levels.

**Fig 9:**
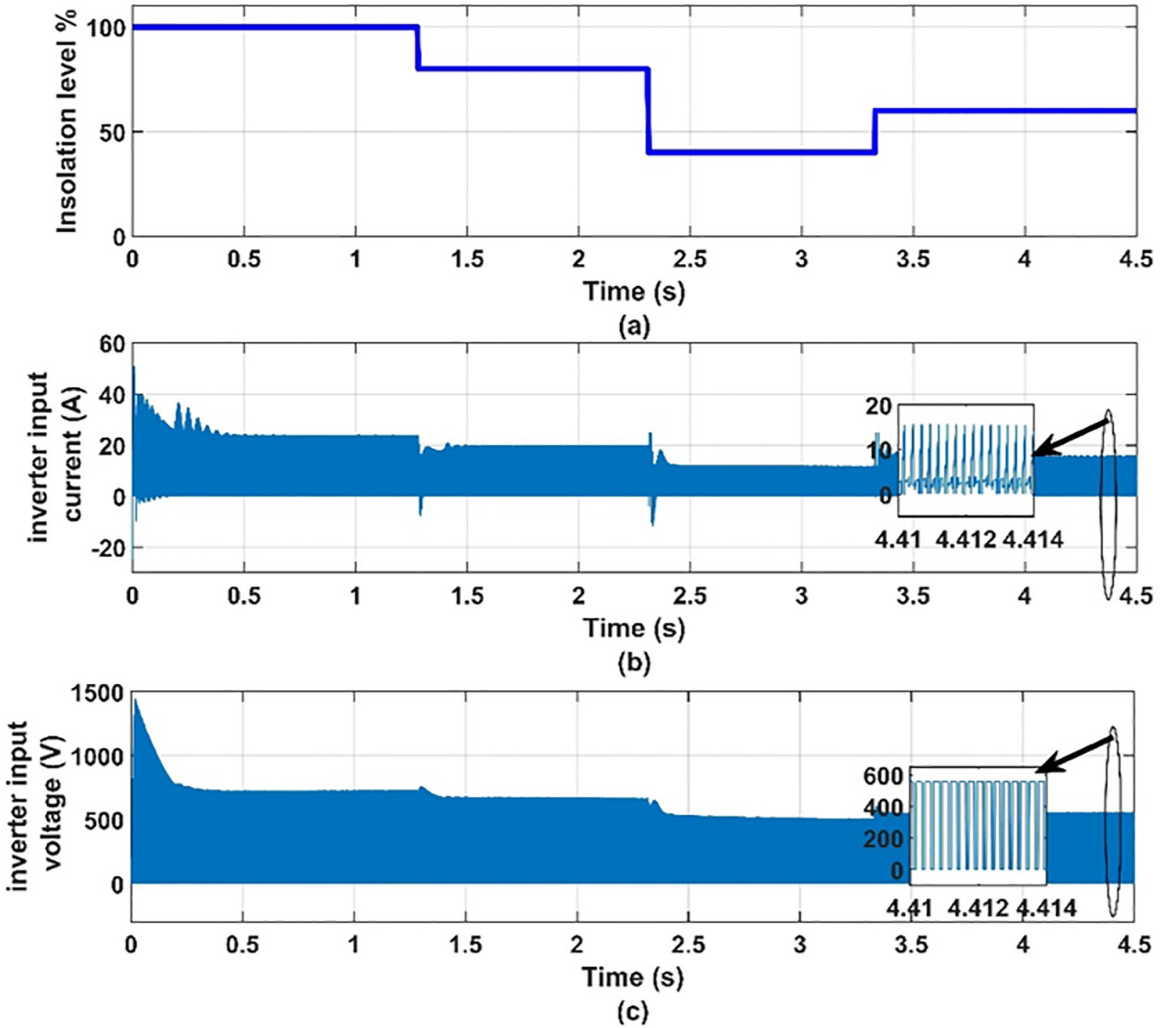
(a) insolation levels, (b) inverter input current and (c) inverter input voltage.

**Fig 10:**
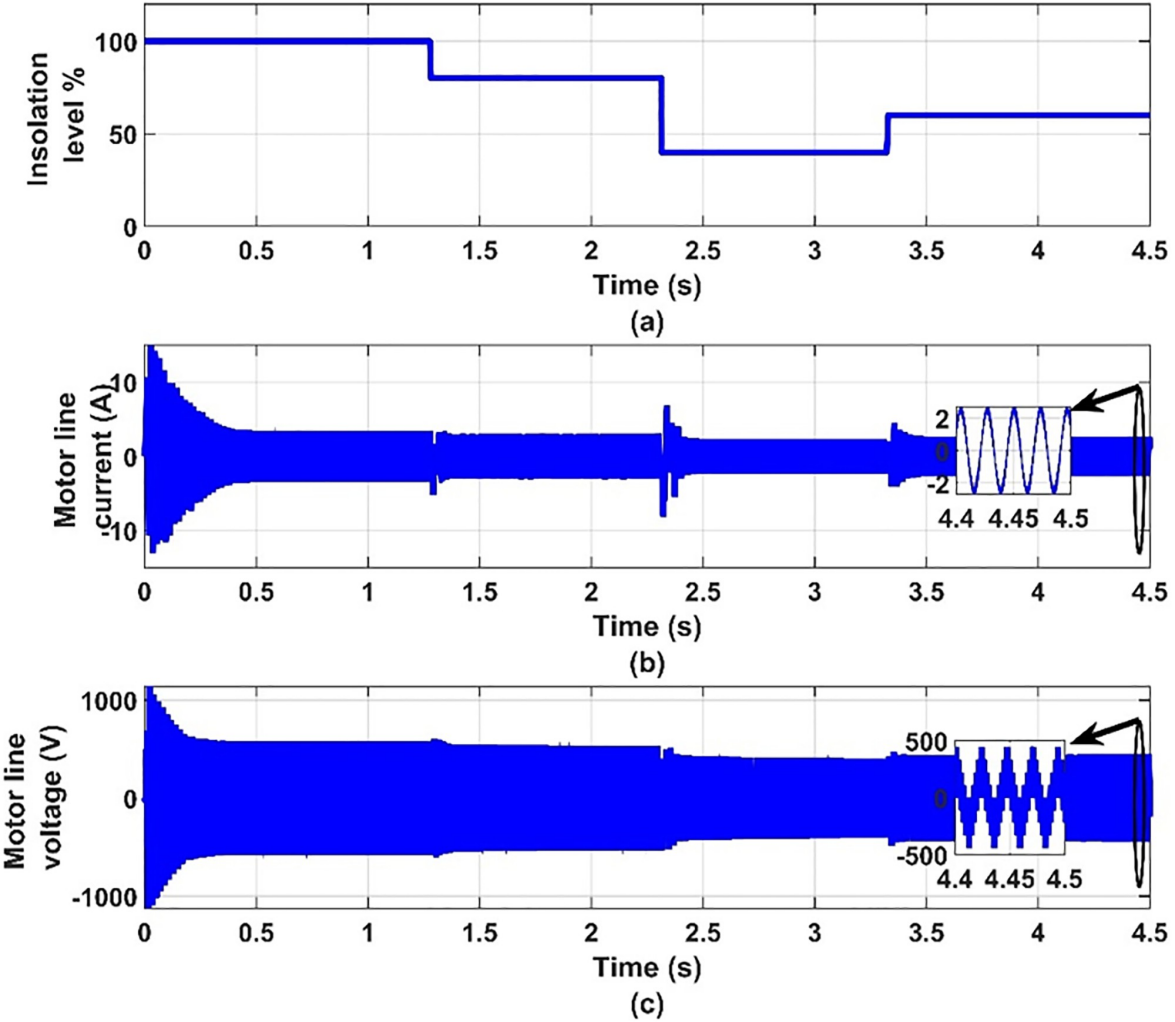
(a) insolation levels, (b) the motor line current and (c) motor line voltage.

**Fig 11:**
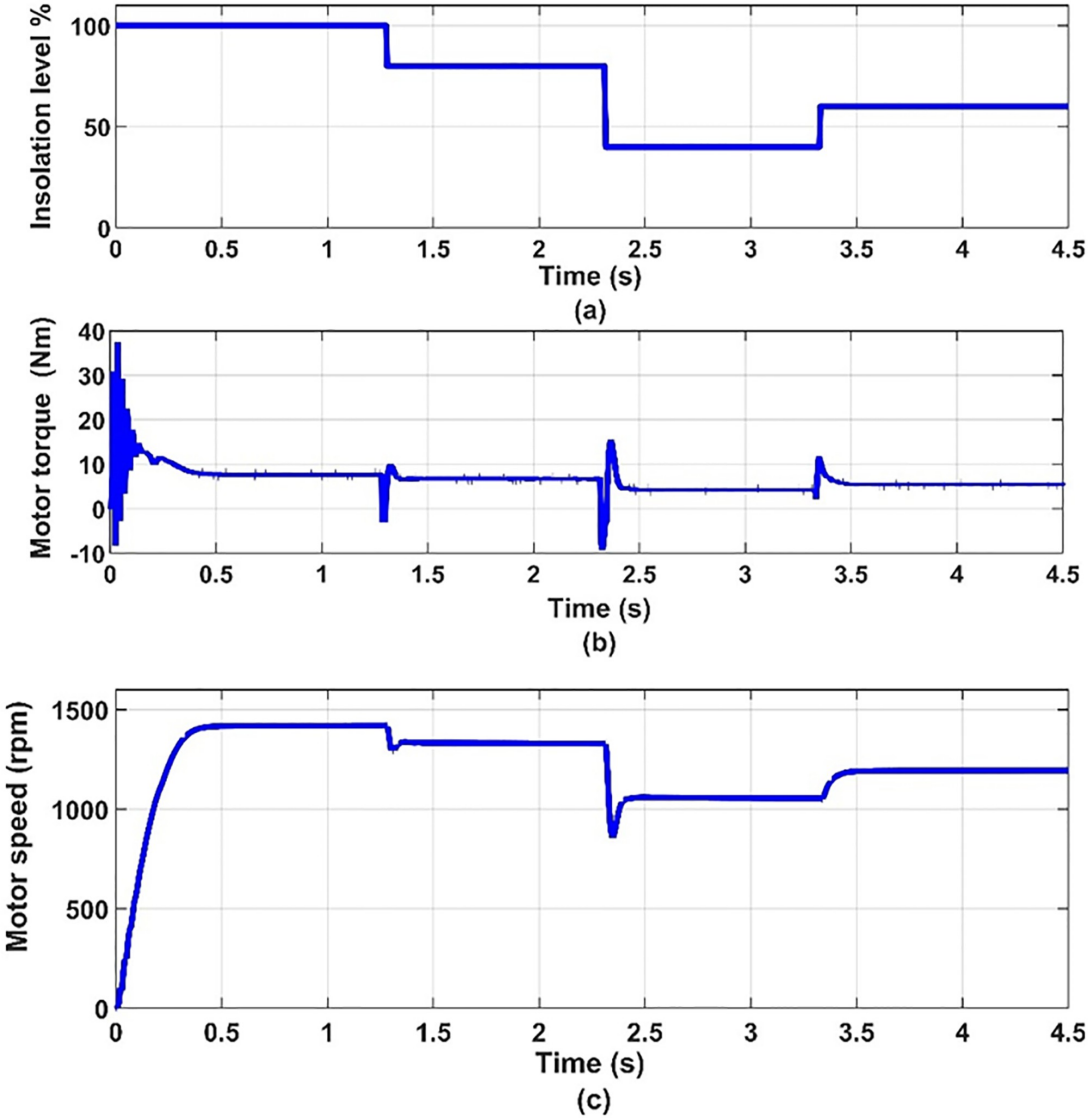
(a) the insolation levels, (a) motor torque and (c) motor speed.

Dynamic results are obtained for the system under consideration for different values of insolation levels, 100%, 80%, 40% and 60% at 25 C° temperature. The inverter switching frequency is taken to be 2 kHz. The five-phase induction motor parameters and the PV-array data are given in appendix. These results are given in Figs 6 to 11 to prove the validity of the proposed method of control.

From Fig 6(a), we find that when the insolation level decreases to 80% and 40%, the frequency of the voltage coming out of the inverter will decrease, as in Fig 6(b), thus reducing the speed of the motor. By applying Eq (20), we find that the power of the motor decreases and thus the pump power decreases. We also find that when the insolation level rises to 60%, the frequency of the voltage coming out of the inverter will increase, and thus the speed of the motor will increase, the power of the motor will increase, and thus the power of the pump will increase.

It can be seen from Fig 7 that when the insolation level decreases, the current of the solar cell decreases and the voltage increases, and thus the power output from it changes. Thus, we find that the photovoltaic array operates at the maximum power point for the characteristics of the photovoltaic array, as shown in Fig 8. It can be seen from Fig 9 that the input voltage and inverter current change with the change in insolation level and contain ripples. It can also be noted from Fig (10) that the motor line current is almost sinusoidal, because of the inductance in the motor windings. It can also be noted from Fig 11 that as the insolation level changes, the operating speed changes in response to reaching the maximum power point.

**Fig 12:**
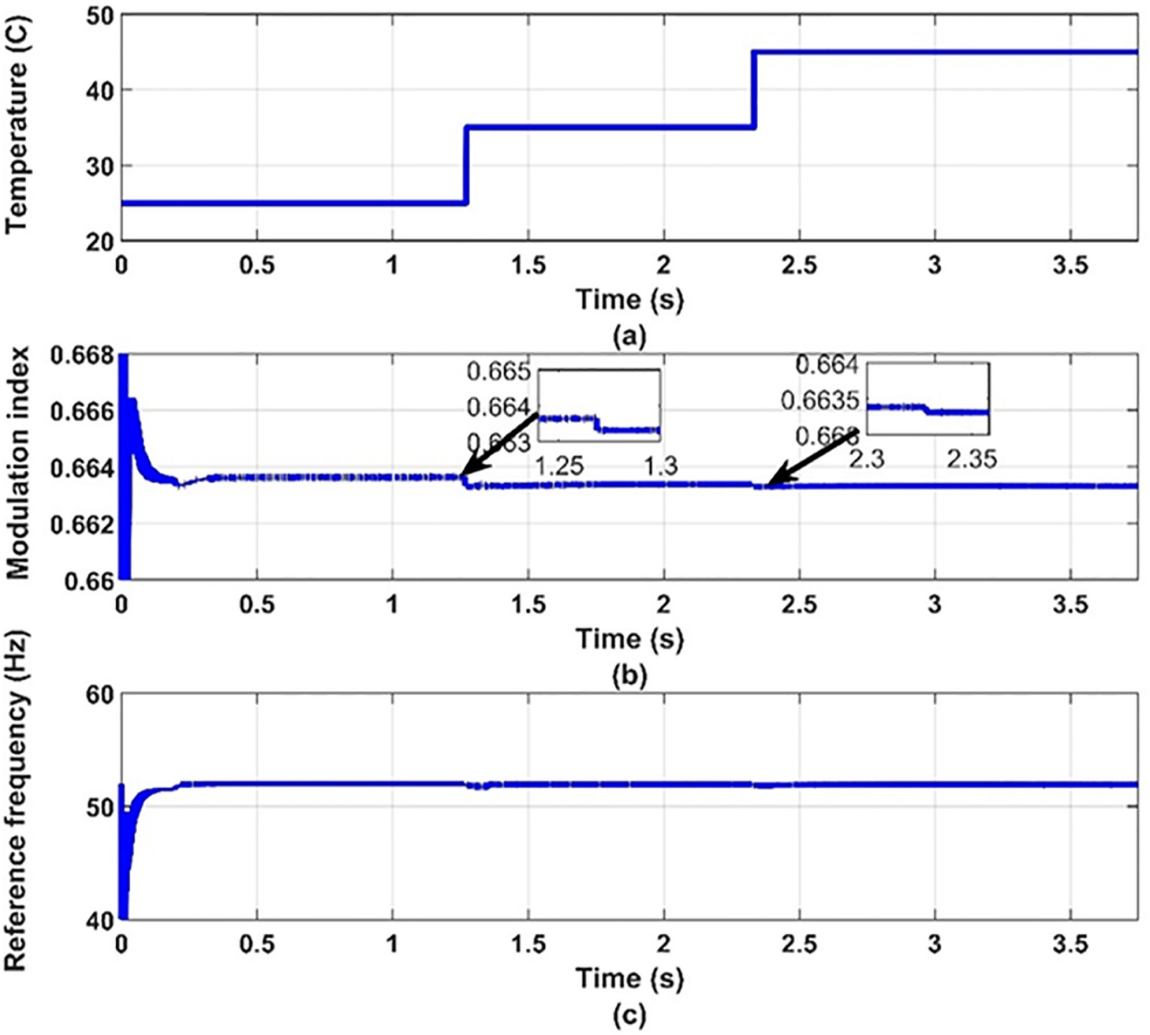
(a) temperature, (b) modulation index and (c) inverter reference frequency.

**Fig 13:**
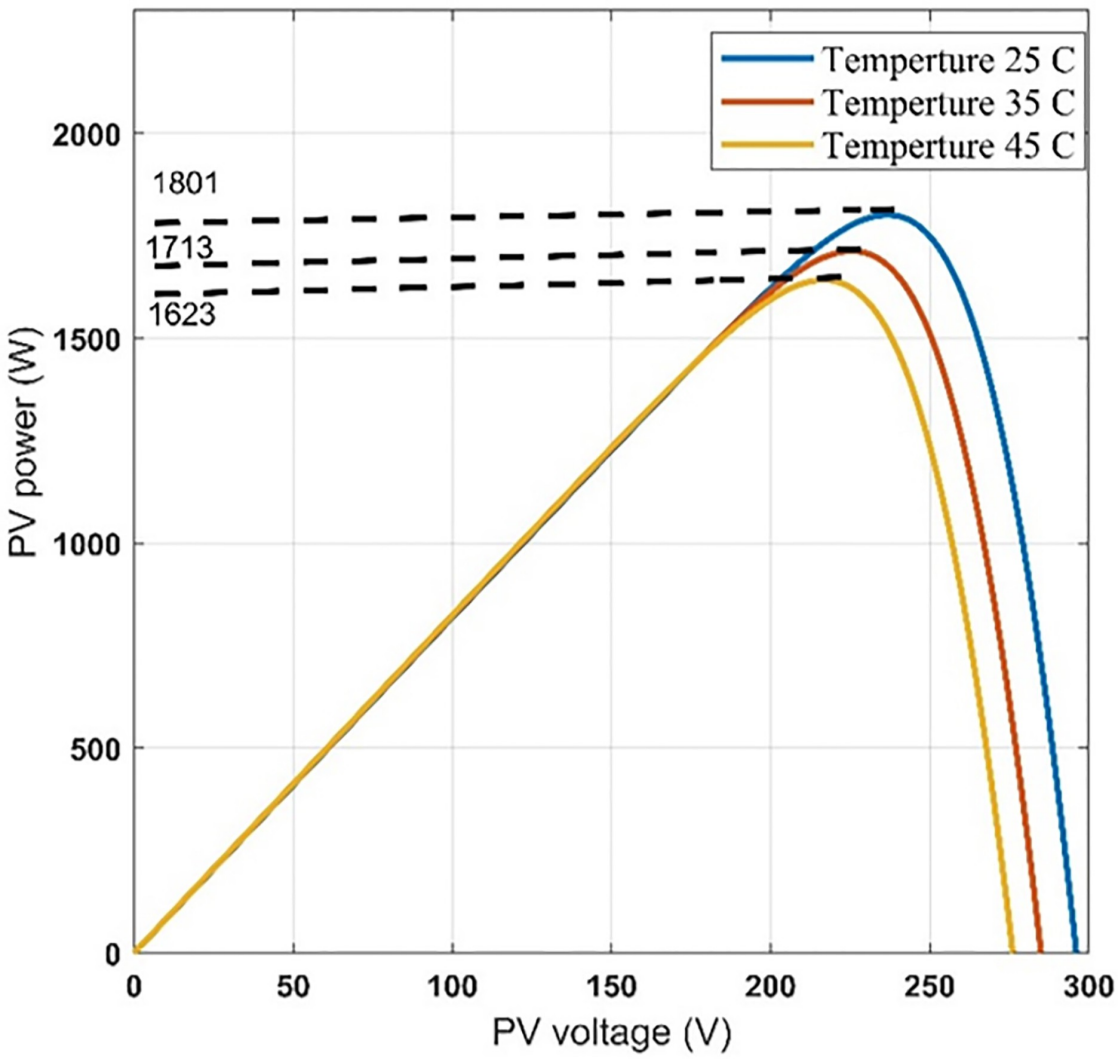
PV-array power-voltage characteristics at different temperatures.

**Fig 14:**
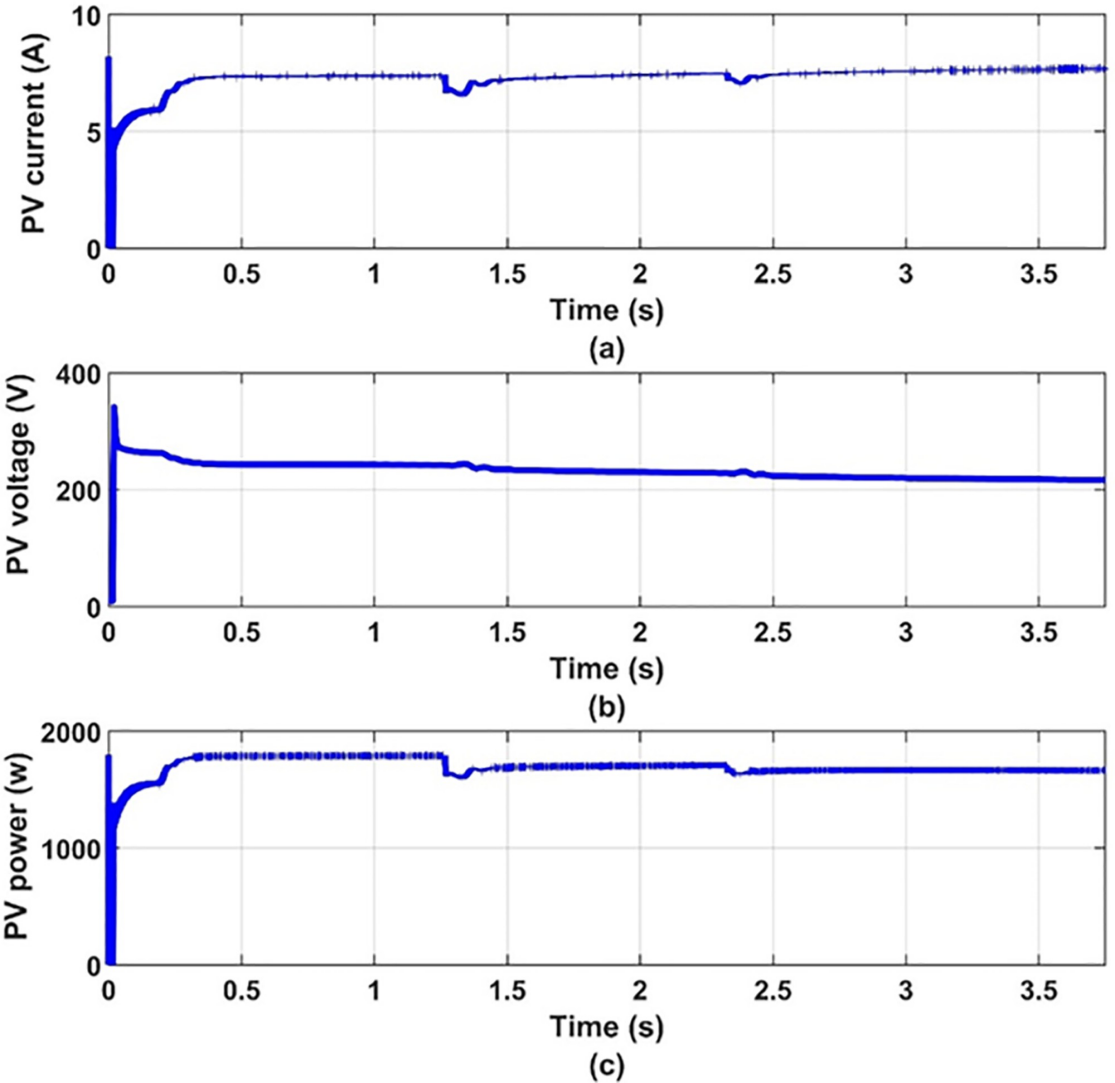
(a) PV-array current, (b) PV-array voltage and (c) PV-array power.

**Fig 15:**
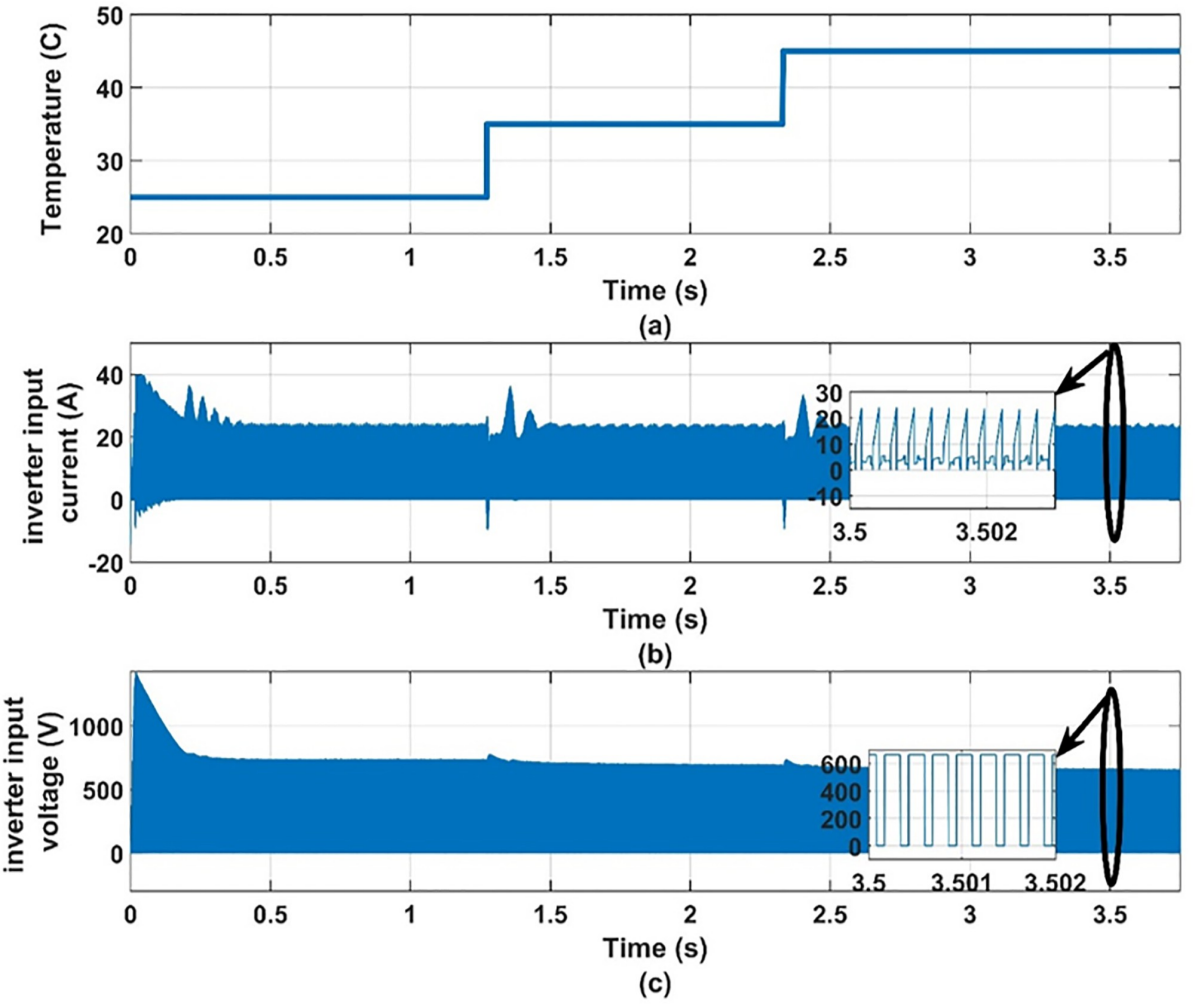
(a) temperatures, (b) inverter input current and (c) inverter input voltage.

**Fig 16:**
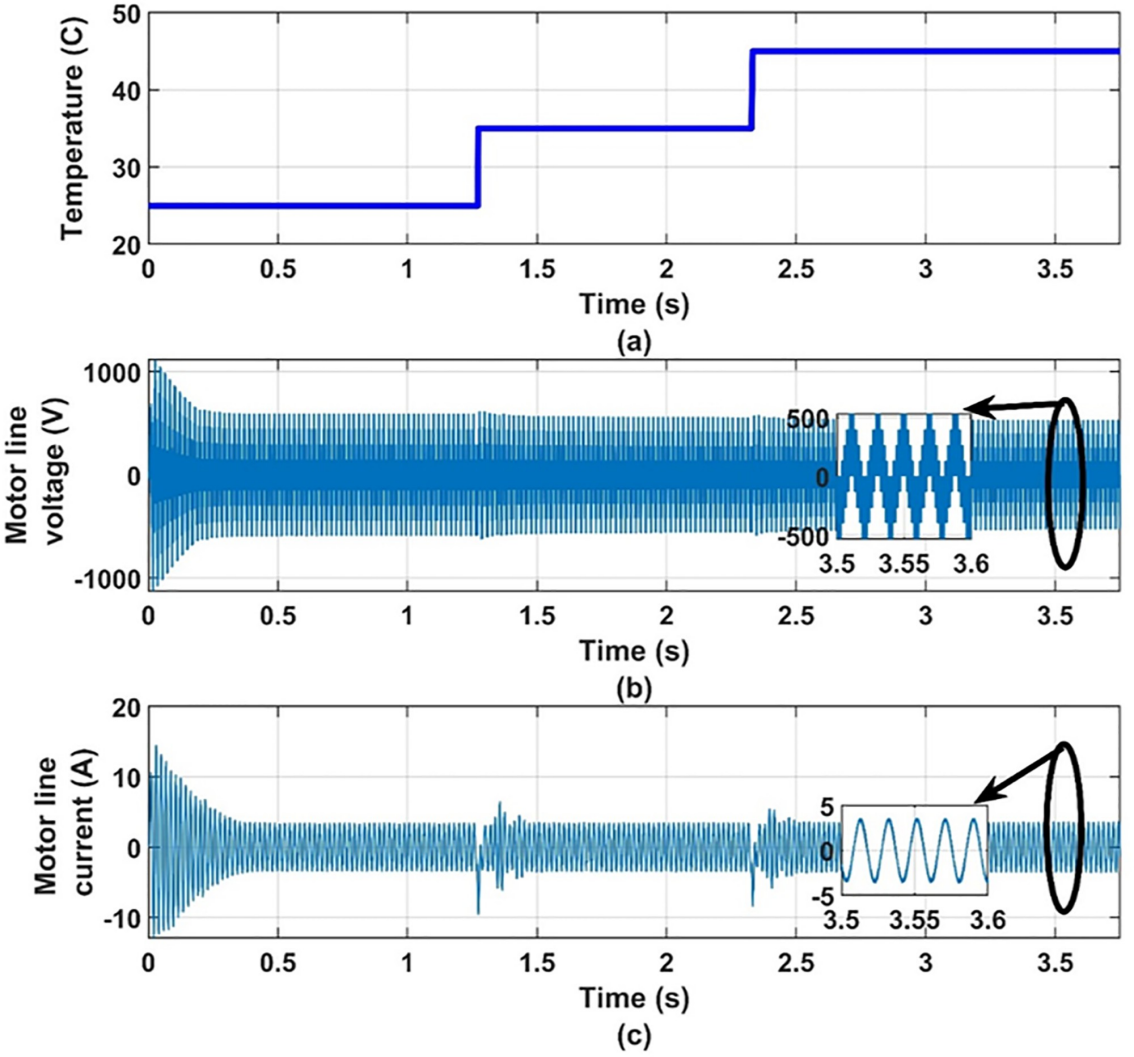
(a) temperatures, (b) motor line current and (c) motor line voltage.

**Fig 17:**
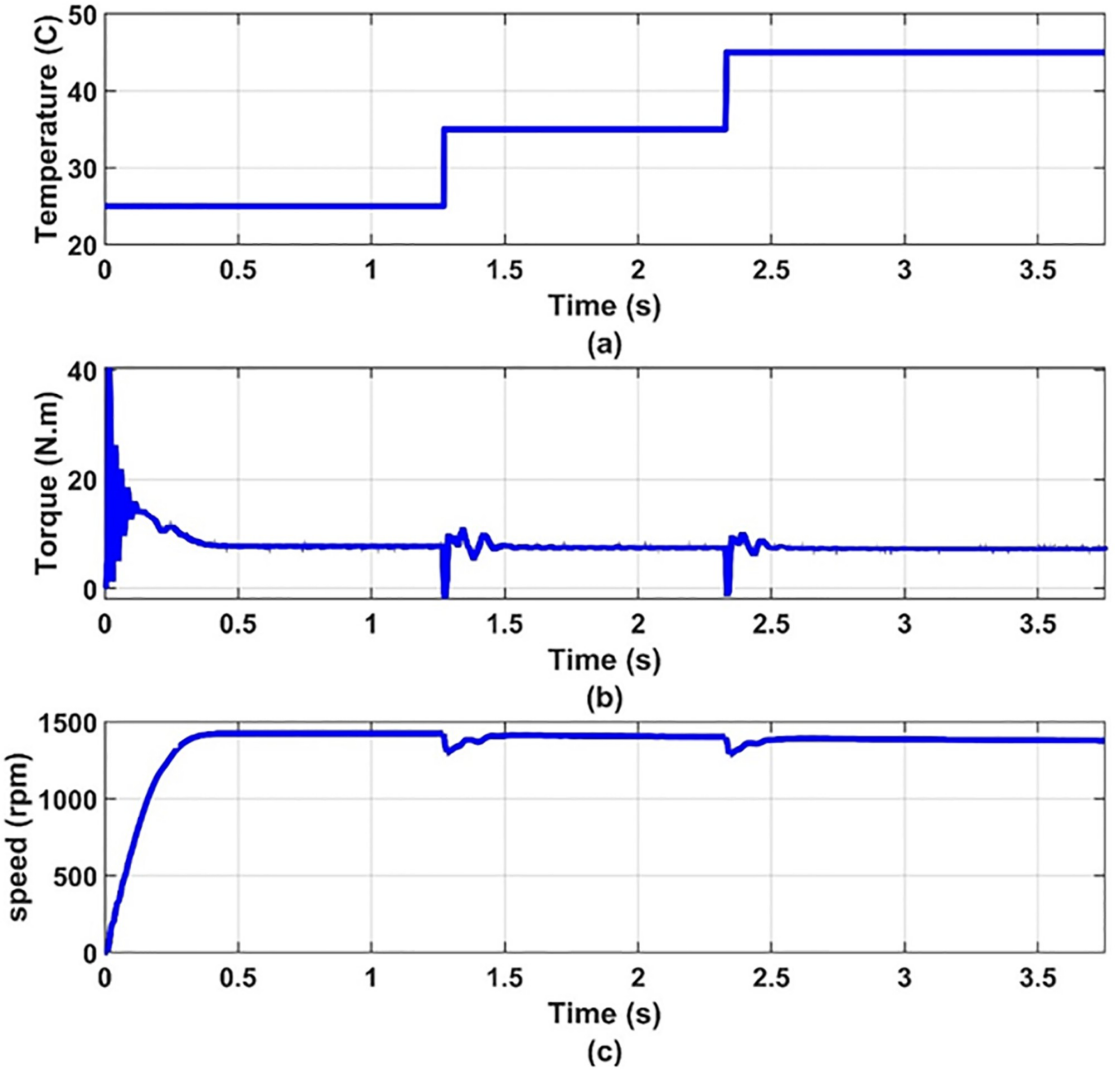
(a) temperatures, (b) motor torque and (c) motor speed.

Another set of dynamic results are obtained for the system under consideration for different values of temperature, 25°C, 35°C, 45°C, at 100% insolation level. The PV array is operated at maximum power point at the different values of temperature. These results are given in Figs 12 to 17 to again prove the validity of the proposed method of control.

It can be noticed from Fig 12 that the effect of changing the temperature on the values of modulation index and the reference frequency is small. This is expected because the effect of temperature on the PV-array maximum power point is small as shown in Fig 13. Therefore, the effect of temperature variation on the other quantities is very small as shown in Figs 14–17, which proves the validity of the proposed method.

## Conclusions

A control method is proposed for solar pumping system based five-phase impedance source inverter and five-phase induction motor. This method is based on controlling the motor speed to control the pump power as the insolation level or temperature change to attain the maximum power extraction from the PV-array. The motor speed is controlled by using an artificial neural network which is trained to provide the desired inverter frequency and modulation index at any insolation level and temperature to attain the maximum PV operating power. The data of the neural network are based on the operation of the induction motor at constant air gap flux and perturb and observe method for maximum power point tracking. Dynamic simulation results are obtained using MATLAB Simulink that proved the validity of the proposed control method.

## Supporting information

S1 Appendix (DOCX)
