# Supplementary material for: MPPT control of a solar pumping system based five-phase impedance source inverter fed induction motor
Source: PLoS One. 2024 Jan 18;19(1):e0295365. doi: 10.1371/journal.pone.0295365 (PMC10796005; doi:10.1371/journal.pone.0295365)

# **Appendix**

**Five-phase induction motor parameters is**


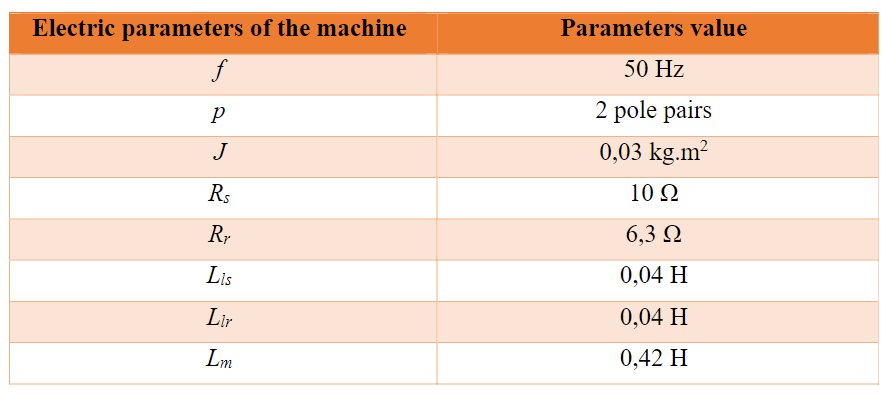


**PV-array parameters**

The PV-module used is the KC200GT. It has a maximum power output of 200 W. The ratings and parameters of the module KC200GT are given below. The PV-array consists of one string which contains nine series connected modules.


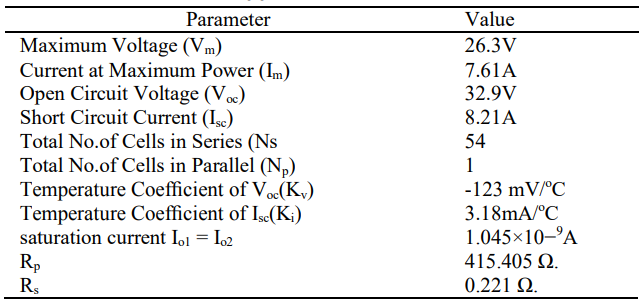

Supplement: S1 Appendix — (DOCX) [file pone.0295365.s001.docx]
